# The Relationship between VO_2_ and Muscle Deoxygenation Kinetics and Upper Body Repeated Sprint Performance in Trained Judokas and Healthy Individuals

**DOI:** 10.3390/ijerph19020861

**Published:** 2022-01-13

**Authors:** André Antunes, Christophe Domingos, Luísa Diniz, Cristina P. Monteiro, Mário C. Espada, Francisco B. Alves, Joana F. Reis

**Affiliations:** 1 Laboratory of Physiology and Biochemistry of Exercise, Faculdade de Motricidade Humana, Universidade de Lisboa, Cruz Quebrada-Dafundo, 1495-761 Lisboa, Portugal; andre.gs.antunes.97@gmail.com (A.A.); luuisamd@hotmail.com (L.D.); cmonteiro@fmh.ulisboa.pt (C.P.M.); falves@fmh.ulisboa.pt (F.B.A.); 2 Life Quality Research Centre, 2040-413 Rio Maior, Portugal; christophedomingos@esdrm.ipsantarem.pt (C.D.); mario.espada@ese.ips.pt (M.C.E.); 3 Interdisciplinary Centre for Human Performance Research (CIPER), Faculdade de Motricidade Humana, Universidade de Lisboa, Cruz Quebrada-Dafundo, 1495-761 Lisboa, Portugal; 4 Polytechnic Institute of Setúbal, School of Education, 2914-514 Setúbal, Portugal

**Keywords:** VO_2_ kinetics, muscle oxygenation, judo, upper body, arm crank, near-infrared spectroscopy, repeated sprint ability

## Abstract

The present study sought to investigate if faster upper body oxygen uptake (VO_2_) and hemoglobin/myoglobin deoxygenation ([HHb]) kinetics during heavy intensity exercise were associated with a greater upper body repeated-sprint ability (RSA) performance in a group of judokas and in a group of individuals of heterogenous fitness level. Eight judokas (JT) and seven untrained healthy participants (UT) completed an incremental step test, two heavy intensity square-wave transitions and an upper body RSA test consisting of four 15 s sprints, with 45 s rest, from which the experimental data were obtained. In the JT group, VO_2_ kinetics, [HHb] kinetics and the parameters determined in the incremental test were not associated with RSA. However, when the two groups were combined, the amplitude of the primary phase VO_2_ and [HHb] were positively associated with the accumulated work in the four sprints (ΣWork). Additionally, maximal aerobic power (MAP), peak VO_2_ and the first ventilatory threshold (VT_1_) showed a positive correlation with ΣWork and an inverse correlation with the decrease in peak power output (Dec-PPO) between the first and fourth sprints. Faster VO_2_ and [HHb] kinetics do not seem to be associated with an increased upper body RSA in JT. However, other variables of aerobic fitness seem to be associated with an increased upper body RSA performance in a group of individuals with heterogeneous fitness level.

## 1. Introduction

Judo is a technically and tactically demanding sport, involving several intermittent efforts of high-intensity activity, interceded by short rest periods [1]. An official, senior-level match may last up to 4 min, and judokas may perform up to seven matches during a tournament, including preliminary rounds, main rounds and finals, all in the same day. This sport is reported to rely heavily on upper body strength and power [2], and it has also been suggested that the high-intensity efforts that occur throughout a match are mainly supported by anaerobic energy systems, while the oxidative energy system may be crucial to the recovery process in between high-intensity efforts and between matches [2].

It has been shown that at the elite level, judo contest winners have a higher activity profile over the course of a match, performing more offensive actions per match (56 offensive actions/match in gold medalists vs. 49 offensive actions/match in silver medalists) [3]. These results highlight that for high-level judo athletes, the ability to perform multiple high-intensity actions over time may be a crucial aspect in determining a contest winner. Therefore, the study of the factors underlying the ability to perform more high-intensity actions over the course of a match in this group of athletes seems to be of relevance.

Many sport activities rely on the ability to repeat several efforts over time. The ability to perform these activities seems to be dependent on the ability to quickly restore phosphocreatine (PCr) stores, ensuring that high rates of muscular work can be sustained over the course of several high-intensity bouts [4]. The ability to restore PCr stores back to near-resting level seems to be dependent on muscular oxidative capacity [5]. Moreover, as these short-duration efforts are repeated over time, the contribution of the oxidative energy system seems to increase [6]. The ability to quickly attain a high rate of ATP resynthesis from oxidative phosphorylation, as expressed by the rate at which oxygen uptake (VO_2_) rises, seems to be an important aspect in delaying the onset of fatigue [7]. As this quick rise is associated with a reduction in oxygen deficit [8], it can contribute to an increased high-intensity exercise tolerance.

The speed at which VO_2_ rises to attain a given value necessary to support the exercise workload can be characterized by the time constant of the primary component of VO_2_ kinetics (τ_phase II_), which represents the time necessary for 63% of the final oxygen uptake response to be complete [9,10,11]. In response to moderate-intensity workloads, the VO_2_ response is characterized by three phases [12,13]: Phase I is the cardiodynamic phase, and corresponds to the phase during which VO_2_ rises as a consequence of increased pulmonary blood flow. Phase II corresponds to the primary component, where VO_2_ rises in an exponential manner, until a steady-state VO_2_ is achieved, corresponding to phase III. The profile of VO_2_ response during phase II seems to closely reflect muscle VO_2_ profile [13,14,15]. During heavy-intensity constant load exercise, the attainment of a steady-state VO_2_ is delayed due to the rise in VO_2_, which exceeds the expected values of VO_2_ based on the VO_2_–exercise intensity relationship established during submaximal (moderate intensity domain) workloads, coinciding with the emergence of a VO_2_ slow component (VO_2__SC_). 

Faster VO_2_ kinetics, characterized by shorter values for τ_phase II_, have been observed in trained individuals [16,17,18,19,20] and have been associated with a smaller decrease in speed over a repeated-sprint ability test (RSA) in a group of soccer players [21]. Moreover, a shorter τ has been associated with longer high-speed running distances in a group of young high-level soccer athletes [22]. Trained individuals have also been shown to have a smaller VO_2__SC_ at a given workload compared to untrained individuals [23,24], which has been associated with an increase in the ability to sustain high-intensity exercise workloads over time at a given absolute workload [25]. Some studies have also shown that upper body trained individuals have faster VO_2_ kinetics compared to untrained subjects [18,19]. To our knowledge, no study to date has sought to understand the characteristics of the response of VO_2_ kinetics during heavy-intensity upper body exercise, nor attempted to establish a relationship between VO_2_ kinetics variables and upper body high-intensity exercise performance in a group of judo athletes.

Near infrared spectroscopy (NIRS) has been used to examine the relative matching of O_2_ delivery with tissue oxygen utilization during constant-workload exercise transitions [26]. The hemoglobin/myoglobin deoxygenation ([HHb]) signal derived from NIRS measurements is reported to reflect the balance between O_2_ delivery and O_2_ utilization and has been used as a non-invasive index of O_2_ extraction from muscle capillaries during exercise [27,28]. 

Given that several variables of aerobic fitness, such as maximal aerobic speed [29] and τ_phase II_ [21], have been associated with increased RSA, we hypothesized that participants with faster upper body VO_2_ and [HHb] kinetics would achieve a higher upper body RSA performance. Therefore, the present study sought to understand if faster VO_2_ and [HHb] kinetics were associated with a higher performance, expressed as a lower decrease in peak power output (PPO) and mean power output (MPO), as well as a higher accumulated work (ΣW), over the course of an upper body RSA test in a group of judo athletes and in a group of healthy individuals of heterogenous fitness level.

## 2. Materials and Methods

Eight male judo athletes (JT) (age 21.1 ± 3.0 years, height 172.3 ± 4.5 cm, body mass 71.5 ± 7.1 kg, triceps skinfold thickness 4.5 ± 0.7 mm) and seven male untrained healthy participants (UT) (age 22.6 ± 1.0 years, height 172.7 ± 4.5 cm, body mass 64.3 ± 5.8 kg, triceps skinfold thickness 6.6 ± 1.6 mm) volunteered to participate in the study. The JT were all black belts, of national (placed in 1st–7th place in the national championships) and international (placed 3rd–9th place in European and World cups) level and had been training (13.1 ± 2.8 year) and competing regularly (6.0 ± 1.3 competitions in the previous year) for at least three years; the UT were not involved in any upper body exercise modalities, although they were all healthy, active (at least 150 min. of physical activity/wk) individuals [30]. 

None of the participants were suffering from any upper body injuries at the time of testing or recovering from any major upper body injury that had occurred in the past 12 months, nor taking any medicine. None of the individuals of JT were cutting weight nor preparing for a major competition at the time that the testing sessions were undertaken.

In order to determine the sample size for the present study, a priori statistical power analysis was performed with G-Power [31] based on the studies of Dupont [21] and McNarry [32], aiming for a power of 85% (alpha = 0.05, two-tailed). The sample size suggested was of 10 individuals for correlations and 7 for each group for the comparisons. Given the strenuous nature of the tests that were undertaken, and that participants could drop out of the study at any time, additional participants were recruited for a total sample of 15 individuals.

All the participants were fully informed of any risks before giving their written informed consent to participate in the study, in accordance with the requirements outlined by the Ethics Committee of the Faculty of Human Kinetics of the University of Lisbon (approval code 42/2021) and in accordance with the Declaration of Helsinki [33]. 

The participants were required to report to the laboratory on three occasions. To avoid circadian rhythm effects, testing occurred at the same time of day, with each session separated by at least 48 h and all testing sessions were completed within 2 weeks. All subjects were required to present themselves in the laboratory with comfortable clothes, in a rested and hydrated state, to refrain from drinking any sort of alcoholic beverages at least 24 h prior to each testing session, and from eating or taking caffeine 3 h prior to each test.

All tests were performed on an electronically braked arm crank ergometer (Lode Angio, Groningen, Netherlands). In their first session, participants performed an incremental step test to determine maximal aerobic power (MAP), peak oxygen uptake (peak VO_2_), first ventilatory threshold (VT_1_) and its respective workload (W_VT_1_). In the second session, participants performed two heavy-intensity square-wave exercise transitions to determine VO_2_ kinetics and [HHb] kinetics of triceps brachii. Each transition began with 3 min of baseline cranking at 0 W, following which the transition workload was imposed. Each square-wave transition was separated by 1 h of passive recovery. In the third session, participants completed a standardized warm-up, followed by an RSA test, consisting of four 15 s upper body all-out sprints, each interceded by 45 s of passive recovery in between. 

### 2.1. Gas Exchange and Muscle Deoxygenation Measurements

Gas exchange variables were collected breath-by-breath with a gas analyzer (MetaMax 3B, Cortex Biophysik, Leipzig, Germany), after calibration according to the manufacturer’s instructions. 

The changes in the [HHb] signal in the local circulation of the long head of the triceps brachii were monitored using a continuous-wave tissue oximeter (NIMO, Nirox, Brescia, Italy) using the NIRS technique. In order to reliably collect the NIRS signal, the local skin of each participant’s upper arm was initially shaved and cleaned. A probe consisting of a photon emitter and a photon receptor, emitting and detecting near-IR beams with three different wavelengths (685 nm, 850 nm and 980 nm), was attached to the skin surface, and secured with tape and then covered with an optically dense elastic bandage in order to minimize movement, prevent loss of near-IR signal and stray light interference, and also to constrain the signal emission-reception site. The signal was sampled at a frequency of 40 Hz. To account for the effects of adipose tissue thickness on the NIRS signal, the skinfold thickness at the site where NIRS probes were placed was measured with a caliper (Slim Guide Caliper, Creative Health, Ann Arbor, MI, USA) and a correction factor was used in the analysis software (Nimo Data Analysis Peak). All NIRS measurements were conducted on the right limb, and [HHb] was monitored during the second and third testing sessions (square-wave transitions and RSA test, respectively). The validity and limitations associated with the measurements obtained via this oximeter have been reviewed by Rovati and associates [34].

### 2.2. Incremental Step Test

Participants performed an incremental exercise test for determination of MAP, peak VO_2_, VT_1_ and W_VT_1_. Participants performed a 3 min step of baseline cranking at 0 W, following which the power was increased 15 W each min (step) until participants reached voluntary exhaustion. The participants were instructed to crank the wheel at the rate of 70 rotations per minute (rpm), grabbing the handles of the ergometer in a standard position, in which they stood upright with their feet shoulder width apart, flat on the floor, and with their shoulder joint levelled with the pedal crank axle. Handle height and ergometer configuration were recorded and reproduced in subsequent tests. The present incremental test protocol’s characteristics were based on the protocols of Koppo and associates [20] and Schneider and associates [35], which also studied upper body VO_2_ kinetics of a group of heterogenous fitness level. Breath-by-breath pulmonary gas-exchange data were collected continuously during the incremental step test. The peak VO_2_ was taken as the highest 30 s average value attained before the participants reached volitional exhaustion. The MAP was defined as the minimal workload which elicited peak VO_2_.

The VT_1_ was estimated by monitoring the ventilatory equivalents for oxygen (VE/VO_2_) and carbon dioxide (VE/VCO_2_), determined by inspection to define the point at which an increase in VE/VO_2_ was observed, with no concomitant increase in VE/VCO_2_ [36]. The workload over which these responses were observed was defined as the W_VT_1_. Throughout the test, heart rate (HR) was monitored continuously (ONRHYTHM 500, Kalenji, France) and the highest HR value observed in the last stage of exercise was registered as peak HR. The workload associated with VT_1_ was used to determine the intensity for the square-wave transitions, which was set at 20%Δ, calculated as W_VT_1_ plus 20% of the difference between the W_VT_1_ and the MAP.

### 2.3. Square-Wave Transitions

The participants performed two square-wave constant workload transitions for the determination of VO_2_ and [HHb] kinetics, with a workload of 20%Δ, corresponding to a heavy-intensity workload. After a 3 min period of baseline cranking at 0 W, the target workload was imposed. Each square-wave transition lasted 6 min and the transitions were separated by 1 h of passive rest. Given the lower exercise tolerance associated with upper body exercise, a 20%Δ workload was chosen, to ensure that the subjects were working in the heavy-intensity exercise domain without incurring excessive fatigue, which would compromise performance in the subsequent square-wave transition, for both groups, and therefore confound the underlying physiological response.

The VO_2_ data were collected breath-by-breath from each transition and were examined to exclude errant breaths and values lying more than 4 standard deviations from the local mean (based on 5 breaths), and subsequently linearly interpolated to provide 1 s values. The data from the two transitions were then time aligned to the start of exercise and averaged to reduce signal noise and enhance the underlying physiological response characteristics [37].

VO_2_ kinetics parameters were calculated by an iterative procedure, minimizing the sum of the residuals, according to the following bi-exponential model:

VO_2_ (*t*) = VO_2baseline_ + A [1 − e^−(*t*−TDp)/^^τp^] + Asc [1 − e^−(*t*−TDsc)/^^τsc^]

where VO_2_ (*t*) represents the absolute VO_2_ at a given time *t*, VO_2__baseline_ represents the mean VO_2_ under unloaded conditions 30 s prior to the work transition; A, TDp, and τ represent the amplitude, time delay, and time constant, of the phase II of the increase in VO_2_ after the onset of exercise, and Asc, TDsc, and τsc represent the amplitude of the slow component, time delay before the onset of, and time constant of the slow component phase of VO_2_ kinetics, respectively [38]. 

The end-exercise VO_2_ was defined as the mean VO_2_ value obtained in the last 30 s of the 6 min constant workload transitions. The first 20 s of VO_2_ data were excluded from the analysis to remove the influence of the cardiodynamic phase on the subsequent response [39]. Because the asymptotic value of the second function is not necessarily reached at the end of the exercise, the amplitude of the slow component was defined as

A′_SC_ = Asc [1 − e^−(*t*e−TDsc/τsc)^]

where *t*e was the time at the end of the exercise bout [20].

Throughout each square-wave transition, the [HHb] signal was monitored in order to provide a non-invasive surrogate of the changes in O_2_ saturation of the hemoglobin/myoglobin in the local circulation of the long head of the triceps brachii. 

The [HHb] data were normalized to resting values, considering the average of the 3 min rest before the unloaded pedaling, and the [HHb] response was characterized according to a monoexponential model, with a timed-delay (TD) at the onset of exercise, followed by an exponential increase [27] until the end of the exercise period:

[HHb] (*t*) = [HHb]_baseline_ + AHHb [1 − e^−(*t*−TDHHb)/τHHb^]

where [HHb] (*t*) represents the [HHb] at a given time *t*, [HHb]_baseline_ represents the 60 s average [HHb] prior to the participant gripping the handles, and A [HHb] and τ [HHb] correspond to the amplitude and time constant of the exponential phase of [HHb] kinetics, respectively. The TD was defined as the time between the onset of exercise and the time at which a first increase in the [HHb] signal was observed [40], which was determined by visual inspection. [HHb] data were fit from the time of initial increase in [HHb] to 180 s. The exponential-like phase of the [HHb] kinetics was also characterized by an “effective” time constant (τ′), which corresponded to the sum of TD and τ [40]. 

### 2.4. Repeated Sprint Exercise

The upper body RSA test consisted of four 15 s all-out sprints, each separated by 45 s of passive rest. Participants performed a 6 min warm-up at 30 W with a cadence of 70 rpm, with three brief sprints (<5 s duration) during the last 3 min of the warm-up. Participants were then given 2 min of rest before commencing the upper RSA test. Thirty seconds before the start of the test, the participants were asked to grip the ergometer handles. Throughout the whole test, the participants were verbally encouraged to give their maximum effort.

The exercise workload was set at 5% of the body mass of each individual [41]. The peak power output (PPO) and mean power output (MPO) attained during each sprint were monitored, and the total work performed (Work) during each sprint was derived as the integral of power output over the 15 s period. 

The 15 s work period was chosen by considering the data reported by Soriano and associates [42] based on the sum of average time it took male judokas to come to grips (8.4 ± 3.1 s), establish a grip and control their opponent (6.1 ± 3.5 s) and execute a throw (1.3 ± 0.5 s). The 45 s rest period duration was chosen as a compromise between what is observed in a typical judo match (2:1 work-to-rest ratio) [1,2], and the typical work density that has been reported in several RSA studies (1:6-8 work-to-rest ratio) [4,6], to ensure that the work period matched what typically occurs throughout a match, as exercise intensity and duration are the main determinants of energy system specificity [43], while allowing participants to sustain the power output over the course of several high-intensity bouts.

A set of variables were computed, in order to characterize overall RSA performance:

Dec-PPO (Decrease in PPO) = PPO 1st sprint − PPO 4th sprint

Dec-MPO (Decrease in MPO) = MPO 1st sprint − MPO 4th sprint

$$\Sigma \mathrm{Work}\;(\mathrm{Accumulated}\;\mathrm{work})=\sum_{1st\;sprint}^{4th\;sprint}\;\mathrm{Work}\;\mathrm{performed}$$

Throughout each sprint, the [HHb] signal was monitored, and the data collected were used to compute the maximal [HHb] attained in each sprint (Max. A [HHb]).

### 2.5. Statistical Analysis

The results are presented as means ± SD. The Shapiro–Wilk test was used to verify the normal distribution of the data for each variable [44]. Unpaired t-tests were used to compare the differences between groups regarding each variable. Pearson product correlations were used to determine the correlation between variables. In order to determine if a linear relationship could be established between a variable of interest and other independent variables, a stepwise regression analysis was performed, using Peak VO_2_, MAP, VT_1__VO_2_, A_phase II_, τ_phase II_, the effective slow component amplitude (A’SC), A [HHb], τ’ [HHb], Max. A [HHb] 1, Max. A [HHb] 2, Max. A [HHb] 3 and Max. A [HHb] 4 as independent variables, and Σ Work as the dependent variable. The collected data regarding each individual variable were analyzed as a whole, considering all participants as a single heterogeneous group, and were analyzed separately by groups. The effect size for the differences between groups was calculated based on the ratio between the difference in the mean values and the weighted pooled SD. The threshold values for Hedges’ effect size (ES, g) statistics were characterized according to the following scale [45]: <0.20 = negligible effect, 0.20–0.49 = small effect, 0.50–0.79 = moderate effect, ≥0.80 = large effect. 

## 3. Results

### 3.1. Incremental Step Test

Mean and standard deviation of the variables obtained by each group of participants in the incremental test are depicted in Table 1.

The JT group displayed higher Peak VO_2_, MAP, W_VT_1_ and 20%Δ than the UT group. A large effect size was observed for Peak VO_2_ (g = 1.8), MAP (g = 3.0), W_VT1 (g = 1.7) and 20%Δ (g = 1.9). VT_1_ _VO_2_ and peak HR were not different between groups.

### 3.2. Square-Wave Transitions

Table 2 shows the VO_2_ kinetics variables obtained by the two groups of participants in the heavy-intensity square-wave transitions.

The JT group presented significantly lower τ_phase II_ and higher τ_SC_ than UT. None of the other VO_2_ kinetics parameters were different between groups. Large effect sizes were observed for τ_phase II_ (g = 1.2) and τ_SC_ (g = 1.2). 

The normalized parameters of the response of [HHb] in the heavy-intensity exercise transitions for the two groups are presented in Table 3.

The JT group presented significantly higher A [HHb] than the untrained participants, whereas τ’ was not significantly different between groups. A large effect size was observed for A [HHb] (g = 1.1)

### 3.3. Upper Body RSA Protocol

The Dec-PPO, Dec-MPO and ΣWork of the RSA test obtained for each group are shown in Table 4.

There were significant differences between groups in the Dec-PPO, with the JT group displaying a lower Dec-PPO over the course of the upper body RSA protocol. Significant differences were also observed between groups in the ΣWork, with a larger mean value of ΣWork being observed in the JT group. A large effect size was observed for Dec-PPO (g= 1.7) and ΣWork (g = 2.5).

### 3.4. Correlations between RSA and the Physiological Variables Obtained in the Square-Wave Transitions and Incremental Step Test 

**Figure 1:**
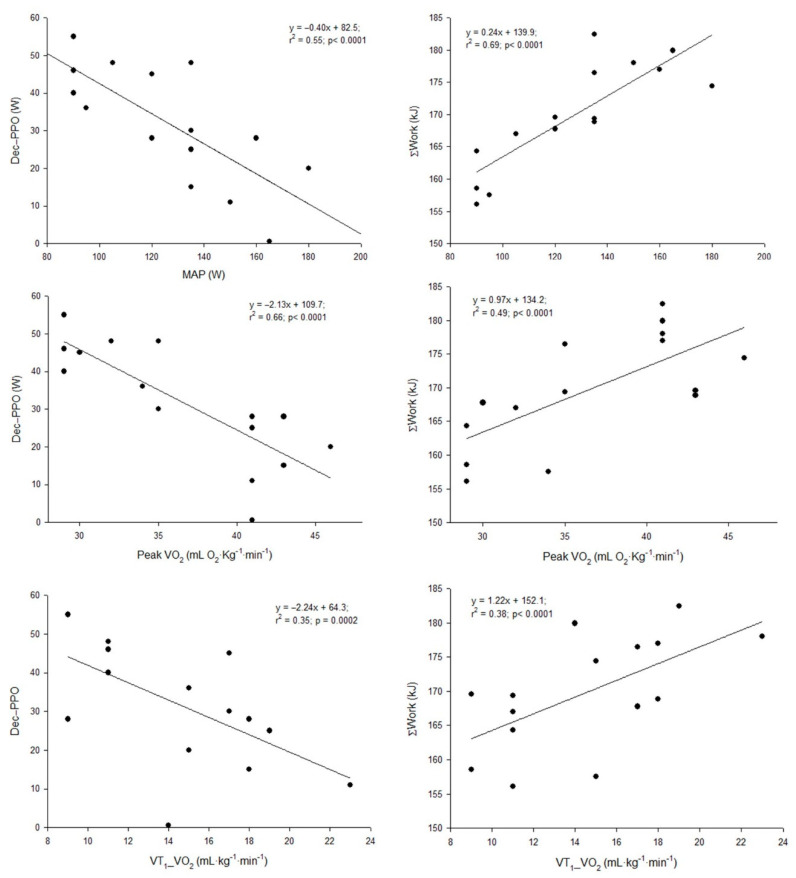
Relationships (correlations) between maximal aerobic power (MAP), peak oxygen consumption (peak VO_2_) and oxygen consumption at the first ventilatory threshold (VT1_VO_2_) achieved in the incremental test and the decrement in peak power output (Dec-PPO) and accumulated work (ΣWork) over the course of the upper body repeated sprint (RSA) test, observed in the group of heterogeneous fitness level (whole sample).

Analysis considering the separate groups did not show any correlation between the performance parameters in the upper body RSA protocol and the parameters determined in the incremental test or square-wave transitions, neither in the JT group nor in the UT group. However, considering the heterogeneous group consisting of the whole sample of participants, significant correlations were found between MAP, peak VO_2_ and VT_1__VO_2_ and Dec-PPO or ΣWork (Figure 1).

**Figure 2:**
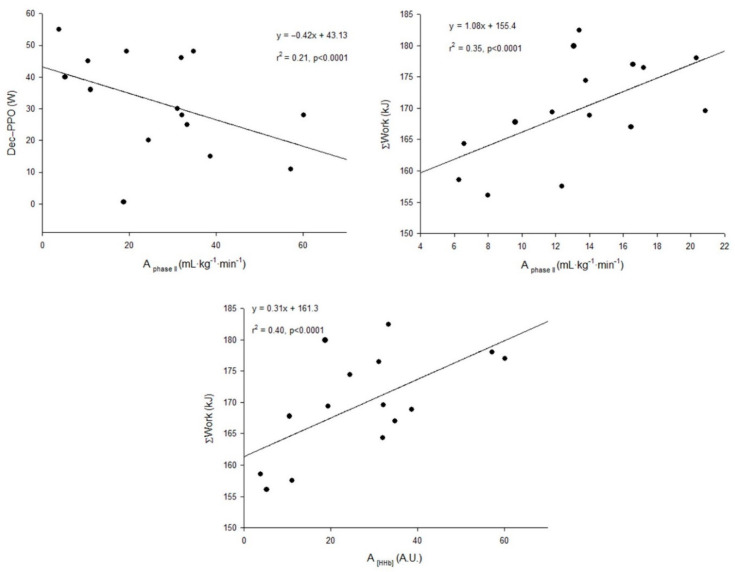
Relationships (correlations) between A of the phase II VO_2_ kinetics, A [HHb] observed during the square-wave transitions and the decrement in peak power output (Dec-PPO) or accumulated work (ΣWork) over the course of the upper body RSA test, observed in the group of heterogeneous fitness level (whole sample).

Additionally, for the UT, a significant correlation was found between A_phase II_ and Dec-PPO or ΣWork and between A [HHb] and ΣWork, as highlighted in Figure 2.

No other significant correlations were observed between any of the performance variables and the VO_2_ kinetics and [HHb] kinetics variables, whether when we consider each group separately or when we analyze the whole sample as a single heterogeneous group.

### 3.5. Predictive Model for ΣWork over the Course of the RSA Protocol

Considering the whole sample as a single heterogeneous group, a significant regression equation was found (F (2.12) = 12.737; *p* < 0.001) with an r^2^ of 0.68, presented in Table 5, for which the main predictors were Peak VO_2_ and Max. A [HHb] 4. The model had a y-intercept at 132.9 kJ, with the ΣWork increasing 0.8 kJ per each unit of increase in Peak VO_2_ and increasing 0.16 kJ per each unit of increase in Max. A [HHb]. These two variables were found to explain 68% of the ΣWork during the RSA protocol. 

When each group was analyzed separately, no significant regression equation to predict ΣWork was found.

## 4. Discussion

To our knowledge, this was the first study to date which analyzed the relationship between parameters of aerobic fitness and upper body RSA performance, both in a heterogeneous sample consisting of trained and untrained participants, and more specifically, in a group of trained judo athletes. The present study revealed that a shorter τ_phase II_ and τ’ [HHb] were not correlated to a lower decrease in PO over the course of an upper body RSA protocol, nor with a higher ΣWork. However, other variables of aerobic fitness, namely Peak VO_2_, MAP, VT_1__ VO_2_, and A of the phase II of VO_2_ kinetics, were inversely correlated with the decrease in PO and directly correlated with the ΣWork over the course of the upper body RSA protocol, in the sample comprising both UT and JT groups. A [HHb] was also directly correlated with a higher ΣWork over the course of the upper body RSA protocol, in the sample comprising both UT and JT groups.

It has been proposed that VO_2_ kinetics influences high-intensity exercise performance [46,47,48,49]. Several authors proposed that faster VO_2_ kinetics, as expressed by a shorter τ_phase II_, are associated with the ability to support a given workload without tapping into O_2_ deficit-related metabolic processes [7] and that faster VO_2_ kinetics are related with faster [PCr] recovery kinetics following exercise [48], two potential aspects that may determine exercise tolerance during repeated high-intensity exercise.

The results observed in the current study indicate that there is no significant correlation between pulmonary τ_phase II_ and upper body RSA performance, namely between τ_phase II_ and the Dec-PPO, Dec-MPO or ΣWork over the course of the four sprints, either when we consider the sample of participants as a single heterogeneous group, or when we analyze the JT separately. These results contradict our main hypothesis, in which we proposed that a shorter τ_phase II_, would be associated with improved RSA performance variables, namely a higher ΣWork and smaller Dec-PPO and Dec-MPO. 

Dupont and associates [21] have previously reported a significant direct correlation between τ_phase II_ and relative decrease in speed and total work performed over the course of an RSA protocol in a group of soccer players. Rampini and associates [49] also found a direct and significant (r = 0.62; *p* < 0.05) association between τ_phase II_ and the relative decrease in sprint speed over the course of six 40 m (20 m run-and-back) shuttle sprints separated by 20 s of passive recovery. 

However, in line with our results, Buchheit [50] found no correlations between RSA performance and τ_phase II_, reporting that stepwise multiple regression analysis showed that mean repeated-sprint time, best sprint time and maximal aerobic speed were the only significant predictors of RSA performance. Accordingly, Christensen and associates [51] also found that τ_phase II_ was not associated with better RSA performance in a group of soccer players, although the changes in τ_phase II_ after a speed-endurance training program were associated with changes in RSA performance. 

The studies mentioned above [21,50,51] involved running activities, utilizing a different set of muscle groups, in a different set of participants, exposed to very different training regimens compared to the individuals involved in the present study. However, they allow us to make some assertions regarding the observed results. The protocol used in the study by Dupont and associates [21] involved significantly more volume, and involved active recovery periods between sprints (15 × 40 m sprints, interceded with 25 s of active recovery), which may have biased the contribution of the aerobic energy system to the total work performed, and therefore the degree of association between RSA performance and τ_phase II_, while Buchheit [50] using a set of lower volume RSA protocols (10 × 30 m; 6 × 2 × 15 m; 6 × 16 m; 6 × 16 m; 20 × 15 m; 6 × 25 m) did not find such correlations. It is possible that an association may only be found between τ_phase II_ and RSA performance involving a high volume of RSA activity, given the increased contribution of the aerobic system as the number of sprints increases [6]. It is possible that an association between τ_phase II_ and RSA performance would be found if a protocol involving a higher volume of sprints had been used. Moreover, the possibility that upper body VO_2_ kinetics variables may play a more important role in judo contests that drag over a longer period of time should also be considered.

The results of the present study indicate that there is an inverse correlation between the MAP attained in the incremental step test and the Dec-PPO for the whole group of participants. Given that MAP is associated with training status [52], these results reveal that participants who are “aerobically” trained to a greater extent display an increased ability to resist decreases in PO over the course of repeated high-intensity exercise. Interestingly, this association was not observed for the JT group. It may be that the MAP of the individuals of the JT group was too similar (low range or spread of values) for any significant correlation to be established. It seems that there is a certain fitness threshold for which this association is valid, and that above this fitness threshold, other variables are more important in determining upper body RSA performance. 

An inverse correlation was also found between the peak VO_2_ and the Dec-PPO and a direct correlation between peak VO_2_ and ΣWork. Similar findings have been reported by other authors [4,6]. The importance of peak VO_2_ to RSA performance seems to be two-fold: (1) Across multiple sprints, aerobic ATP provision progressively increases such that aerobic metabolism may contribute as much as 40% of the total energy supply during the final repetitions of an RSA protocol [53]; (2) Enhanced oxygen delivery to muscles post-exercise potentially accelerates the rate of PCr resynthesis, an oxygen-dependent process [53,54], facilitating a faster recovery from high-intensity exercise.

Bishop and associates [55] observed a significant negative correlation (r = −0.50; *p* < 0.05) between VO_2__max_ and % decrease in work over the course of 5 x 6 s sprints in a group of female basketball athletes. The authors proposed that athletes with greater VO_2__max_ would be able to achieve a higher VO_2_ rate throughout each sprint, reducing the contribution of substrate-level phosphorylation to ATP resynthesis, and therefore allowing more work to be done over the course of the RSA protocol [56]. Aguiar and associates [57] also reported a significant negative correlation (r = −0.58, *p* < 0.05) between VO_2__max_ and the decrease in performance over the course of an RSA protocol (10 × 35 m sprints, 20 s recovery between sprints) in a heterogeneous group composed of endurance runners, sprinters and healthy individuals. Collectively, these results seem to emphasize the relationship between VO_2max_ and the ability to maintain a high power output over the course of several RSA efforts. This observed association between VO_2__max_ and the decrease in work capacity over the course of an RSA task may also be associated with a higher cardiac output (Q) and subsequent increase in muscle blood flow, which may aid post-exercise recovery [58].

Furthermore, since a positive correlation was found between A [HHb] and ΣWork, it seems that repeated sprint performance is enhanced in individuals with higher oxygen extraction during heavy intensity exercise. The NIRS-derived [HHb] signal has been considered to reflect the ratio between muscle O_2_ delivery and demand, and therefore has been considered an index of muscle O_2_ extraction [27,59]. A higher A [HHb] has been associated with a greater muscle oxygen extraction following the onset of exercise [60,61] and has been shown to increase following training [62]. This indicates that a greater oxygen extraction at the onset of exercise and in repeated sprints may have an important role in the ability to maintain a constant performance over the course of several upper body high-intensity efforts. 

Moreover, peak VO_2_ and maximal [HHb] achieved in the fourth sprint were found to be significant predictors of ΣWork over the course of the four sprints, which seems to indicate that these aerobic fitness variables contribute to performance in repeated sprints. Specifically, both central and peripheral determinants of oxygen uptake contribute to performance during high intensity efforts where the anaerobic component is predominant. These associations were not observed when we consider the JT group separately, probably due to the more homogeneous response in these parameters in this restricted sample. In light of the observed results, it seems that aerobic fitness variables are associated with and increased upper body RSA performance in a group of individuals of heterogenous fitness. However, it is possible that once a certain level of upper body aerobic fitness is attained, other physiological and fitness variables may play a more important role in determining upper body RSA performance. 

Complementary to this, the present study seems to indicate that there are significant differences between JT and UT participants in regard to upper body VO_2_ kinetics parameters. No previous study has reported upper body VO_2_ kinetics parameters in a group of JT. The τ_phase II_ values observed in this group of athletes are similar to those found by Koppo and associates [20] in a group of physically active males (τ_phase II_ = 48 ± 12 s). The values of VO_2_ kinetics parameters for the group of UT participants observed in this study are similar to those reported by Schneider and associates [35] in a group of untrained participants (τ_phase II_ = 66 ± 3 s). Both mentioned studies analyzed the VO_2_ kinetics response across the same exercise intensity range that was used in the present study. By comparison, the τ_phase II_ values observed by Invernizzi and associates [18] in a group of specifically upper body trained participants (elite competitive swimmers; τ_phase II_ = 34.3 ± 8.5 s), determined in an arm crank ergometer test, were much shorter than those which were observed in the JT group. 

It is possible that judo-specific training may have induced sufficient training adaptations that resulted in a faster VO_2_ kinetics response to exercise relative to untrained participants. However, given that judo-specific drills involve a different skeletal muscle function regimen, where isometric muscular actions of the upper body are emphasized, and muscle actions are performed in an intermittent way, the physiological adaptations that occur may involve very different mechanisms than those which are associated with the performance of high-volume, continuous exercise training of moderate–heavy exercise intensity, typical of swimming. This may also explain the similar results observed in the upper body VO_2_ kinetics in the JT group compared to a group of physically active males [20]. 

Several studies have observed that different training programs, performed at different training intensities have the potential to induce adaptations compatible with shorter τ_phase II_ of VO_2_ kinetics. Studies have observed improvements in VO_2_ kinetics with training protocols ranging from low-intensity work at 60% VO_2_ max. [62] to sprint-interval training performed at supramaximal intensities [60]. Nevertheless, these observations have been reported for studies involving dynamic, running or cycling exercise, which involve a different set of muscle groups and muscle action regimen compared to judo-specific training. As it has already been noted, judo-specific modalities seem to rely more on upper body musculature [2]. Given that upper body exercise has been associated with different hemodynamic [63] and metabolic responses [64], physiological adaptations may vary considerably compared to other forms of exercise. 

McNeil and associates [65] have observed that performing isometric dorsiflexions at 100% of the maximal voluntary contraction (MVC) resulted in significant decreases in NIRS-derived tissue oxygenation compared to performing isometric dorsiflexions at 30% of MVC. Even though these authors reported no significant changes in tibial artery mean blood flow during the course of 60 s of sustained contraction, they suggested that the capillary mean blood flow might have been severely compromised over the course of the sustained exercise, and that this may have compromised tissue oxygenation dynamics throughout the 100% MVC exercise periods [66]. Given that, over the course of judo training and competition drills, athletes are likely to be exposed to similar conditions, muscle oxygen uptake dynamics might be compromised, and in turn, this may influence the type of physiological adaptations that take place.

The current study presents some limitations, which may limit the degree to which we can generalize the observations that were made. The sample size was relatively small, which affects the statistical meaningfulness of the observed results, as well as the degree to which we can extrapolate our conclusions. Nonetheless, it satisfies the requirements of statistical power determined a priori. Moreover, the athletes that participated in the present study were mostly national-level athletes, although it included some international-level athletes, and was therefore quite heterogenous. It is possible that different associations may have been found if the study had included judo athletes with a higher performance level, and therefore, further conclusions might be drawn regarding the physiological and fitness parameters which may be associated with upper body RSA performance in this group of athletes. 

Furthermore, accessing body composition variables (% fat and fat-free mass), and other physical fitness variables (maximal upper body strength, anaerobic power) could have provided further insight regarding the determinants of upper body RSA performance and help explain the differences observed between groups. Although we designed the RSA test according to the observations reported by Soriano and associates [42], where the sum of time to kumi kata (8.4 s), kumi kata (6.1 s) and throwing time (1.3 s) corresponded to approximately 15 s, the duration chosen for the working periods, we recognize that the number of sprints may have been insufficient to match the number of sequences of attacks observed in more prolonged judo matches. It should also be noted that these tests were undertaken in an arm-crank ergometer, which is a general form of upper body dynamic exercise, and therefore the observed performance achieved by each individual in this task bears little resemblance to what actually happens over the course of a judo match. However, given that judo, as a grappling sport, relies heavily on the upper body musculature, a greater ability to preserve the work capacity of these muscle groups throughout a match seems to be a relevant aspect for potentially achieving a greater performance in the context of a judo match.

No studies to date had attempted to study the fitness variables underlying upper body RSA performance, particularly involving judo athletes. Therefore, the present study provides valuable information for further research regarding the variables that determine upper body RSA performance, particularly in this group of athletes, which may shed light regarding the factors that contribute to maintaining a high activity/attack profile over the course of a match.

Future research should consider using different repeated-sprint protocols with more repetitions and possibly a different work:rest period in order to reveal which factors may be associated with improved repeated-sprint ability under different match conditions. Moreover, future studies should include a larger sample of individuals, whether in a group of judokas of heterogenous fitness level/competitive status or in specific groups (international/elite vs. national level athletes), in order to better understand the factors that may determine RSA performance capacity in different groups. Future studies should also seek to include female individuals, in order to understand if different fitness variables influence upper body RSA performance for both sexes. 

### Practical Applications for Coaches

In lower-level athletes, developing a higher upper body aerobic fitness through higher volume general or specific exercises/drills would benefit their ability to maintain a higher performance over the course of the several high-intensity sequences of activity that take place during a match. However, for higher level athletes who already have reasonable upper body aerobic fitness, it may be more pertinent to devote time to developing other fitness variables in order to improve the ability to sustain a higher performance over the course of a judo match. The variables that ought to be developed in order to improve upper body RSA ability in higher-level judo athletes warrant further investigations.

## 5. Conclusions

The main conclusions of the present study are the following: (1) There seems to be no significant correlation between τ_phase II_ or τ’ [HHb] and upper body RSA performance in a group of judokas or in a group of subjects with a heterogeneous fitness level; (2) No significant correlations seem to exist between peak VO_2_, MAP or VT_1__VO_2_ and upper body RSA variables in a group of trained judokas; (3) Judokas displayed significantly faster VO_2_ kinetics and higher muscle oxygen extraction in heavy-intensity exercise than untrained participants and; (4) There seems to be a positive association between MAP, peak VO_2_, VT_1__VO_2_, A_phase II_ and A [HHb] and ΣWork over the course of an upper body RSA task in a group of participants of heterogeneous fitness level. Therefore, aerobic fitness variables seem to play an important role in upper body RSA performance in individuals with a heterogenous fitness level.

## Figures and Tables

**Table 1:** Physiological responses attained by UT and JT participants in the incremental step test.

Variables	UT	JT	*p*
Peak VO_2_ (mL·kg^−1^·min^−1^)	32.3 ± 5.1	40.4 ± 3.7	0.004 *
MAP (W)	101.4 ± 13.8	149.3 ± 17.4	<0.001 *
VT_1__VO_2_ (mL·kg^−1^·min^−1^)	11.9 ± 1.1	16.9 ± 1.3	0.289
W_VT_1_ (W)	42.9 ± 20.2	69.4 ± 11.2	0.007 *
20%Δ (W)	57.1 ± 18.5	86.5 ± 11.7	0.003 *
Peak HR (beats/min)	172.4 ± 11.4	177.0 ± 6.4	0.349

UT, untrained participants; JT, judo athletes; Peak VO_2_, peak oxygen consumption; MAP, maximal aerobic power; VT_1__VO_2_, oxygen consumption rate at the onset of the first ventilatory threshold; VT_1__W, workload at the onset of the first ventilatory threshold; 20% ΔW, Workload corresponding to the sum of VT_1__W plus 20% of the difference between the MAP and VT_1__W; Peak HR, Peak heart rate achieved during the incremental test; * Significant differences between groups for *p* < 0.05.

**Table 2:** VO_2_ kinetics parameters in the heavy-intensity square-wave transitions for each group.

Variables	UT	JT	*p*
VO_2baseline_ (mL·kg^−1^·min^−1^)	9.4 ± 1.2	9.1 ± 1.1	0.627
A_phase II_ (mL·kg^−1^·min^−1^)	11.5 ± 5.5	15.1 ± 2.8	0.128
TD_phase II_ (s)	11.4 ± 9.2	10.5 ± 8.0	0.850
τ_phase II_ (s)	61.6 ± 8.2	47.5 ± 13.4	0.032 *
A’_SC_ (mL·kg^−1^·min^−1^)	2.6 ± 0.7	4.9 ± 3.4	0.097
TD_SC_ (s)	204.4 ± 74.9	175.6 ± 49.0	0.388
τ_SC_ (s)	53.6 ± 26.5	102.2 ± 52.1	0.045 *
EE VO_2_ (mL·kg^−1^·min^−1^)	23.9 ± 6.1	28.4 ± 3.4	0.094
A’_SC_/EE VO_2_	0.1 ± 0.1	0.2 ± 0.1	0.191
Sum of residuals	533.2 ± 222.9	369.0 ± 185.6	0.141

UT, untrained participants; JT, judo athletes; VO_2baseline_, baseline oxygen consumption rate; A_phase II_, Amplitude of the primary phase; τ_phase II_, Time constant of the primary phase; TD_phase II_, Time delay of the primary phase; Asc, Amplitude of the slow component phase; A’sc, Effective amplitude of the slow component; TD_SC_, Time delay of the slow component phase; τ_sc_, Time constant of the slow component phase; EE VO_2_, oxygen uptake rate observed at the end of the square-wave transitions; A’SC/EE VO_2_, Effective amplitude of the slow component relative to the oxygen consumption rate observed at the end of the square-wave transitions; Sum of residuals, Discrepancy in a dataset that is not explained by the model. * Significant differences between groups for *p* < 0.05.

**Table 3:** Observed [HHb] kinetics during the heavy-intensity exercise square-wave transitions.

Variables	UT	JT	*p*
τ’ [HHb] (s)	36.6 ± 17.1	36.2 ± 10.7	0.587
A [HHb] (A.U.)	18.5 ± 13.8	35.4 ± 15.9	0.049 *

τ’ [HHb], Effective time constant of [HHb] kinetics; A [HHb], Amplitude of response of hemoglobin/myoglobin deoxygenation. * Significant differences between groups for *p* < 0.05.

**Table 4:** Variables of RSA for the two groups of participants.

Variables	UT	JT	*p*
Dec-PPO (W)	42.6 ± 8.8	22.1 ± 14.4	0.006 *
Dec-MPO (W)	14.2 ± 12.0	9.4 ± 9.6	0.406
ΣWork (KJ)	163.0 ± 5.5	175.8 ± 4.8	<0.001 *

Dec-PPO, decrease in the peak power output between the first and fourth sprint, Dec-MPO, decrease in mean power output between the first and fourth sprint; ΣWork, accumulated work in the RSA test. * Significantly different from UT values for *p* < 0.05.

**Table 5:** Predictors of accumulated work over the course of the upper body repeated sprint test for the whole group of participants.

Accumulated Work over the Course of the Upper Body RSA Protocol (kJ)	R	R^2^	Adj. R^2^	SEE	*p*
ΣWork (kJ) = 132.9 + 0.8 Peak VO_2_ + 0.16 Max. A [HHb] 4	0.82	0.68	0.63	5.1	0.001

Peak VO_2_, the highest 30 s average VO_2_ attained over the course of the incremental test, Max. A [HHb] 4, the maximal [HHb] achieved in the fourth repetition of the upper body RSA test. All other variables were excluded from the model.

## Data Availability

The data that support the findings of this study are available from the corresponding author upon reasonable request.
